# Interferon gamma induced-ACSL5 shapes the lipidome of kidney tubular cells

**DOI:** 10.1016/j.isci.2025.112742

**Published:** 2025-05-23

**Authors:** Virginie Poindessous, Julio L. Sampaio, Lydia Bouidghaghen, Ivan Nemazanyy, Alexandre Pallet, Maarten Naesens, Thibaut Vaulet, Dany Anglicheau, Nicolas Pallet

**Affiliations:** 1 Centre de Recherche des Cordeliers, INSERM UMRS1138, Université Paris Cité, Paris, France; 2 CurieCoreTech Metabolomics and Lipidomics Technology Platform, Institut Curie, Paris, France; 3 Platform for Metabolic Analyses, Structure Fédérative de Recherche Necker, INSERM US24/CNRS UMS 3633, Paris, France; 4 Department of Microbiology, Immunology and Transplantation, Nephrology and Kidney Transplantation Research Group, KU Leuven, Leuven, Belgium; 5 Department of Kidney and Metabolic Diseases, Transplantation and Clinical Immunology, Necker Hospital, Assistance Publique - Hôpitaux de Paris Université Paris Cité, Paris, France; 6 Department of Nephrology, Georges Pompidou European Hospital, Assistance Publique Hôpitaux de Paris, Université Paris Cité, Paris, France; 7 Department of Clinical Chemistry, Georges Pompidou European Hospital, Assistance Publique Hôpitaux de Paris, Université Paris Cité, Paris, France

**Keywords:** Integrative aspects of cell biology, Lipidomics, Metabolomics, Transcriptomics

## Abstract

Acyl-CoA synthetase long-chain family (ACSL) enzymes are critical in the activation of long-chain fatty acid. To determine the regulatory mechanisms of ACSL5 and its biological functions within the kidney tubule, we generated transcriptomic, metabolomic, and lipidomic data from experimental models and patient cohorts. We show that ACSL5 is a constituent of a gamma interferon-related gene signature linked to rejection in kidney transplant recipients and the urinary metabolome of kidney transplant recipients who experienced rejection exhibited a deficiency in ACSL5 substrates. We demonstrate that ACSL5 expression is induced in kidney tubular cells in response to IRF-1 signaling, and that it is involved in maintaining ATP production and cell viability and influences their lipid composition, reducing the accumulation of ceramides and the contents in glycerolipids. Thus, modulation of the activity of ACSL5 could impact tubular cell energy metabolism and lipid composition, with a clinical impact in response to kidney allograft injury.

## Highlights

• ACSL5 expression is induced in kidney allografts with T cell-mediated rejection
• ACSL5 is upregulated in response to IRF1 signaling in kidney tubule cells
• ACSL5 is involved in maintaining ATP production and cell viability
• ACLS5 shapes cellular lipid composition, reducing ceramide, and glycerolipid accumulation

## Graphical abstract

**Figure:**
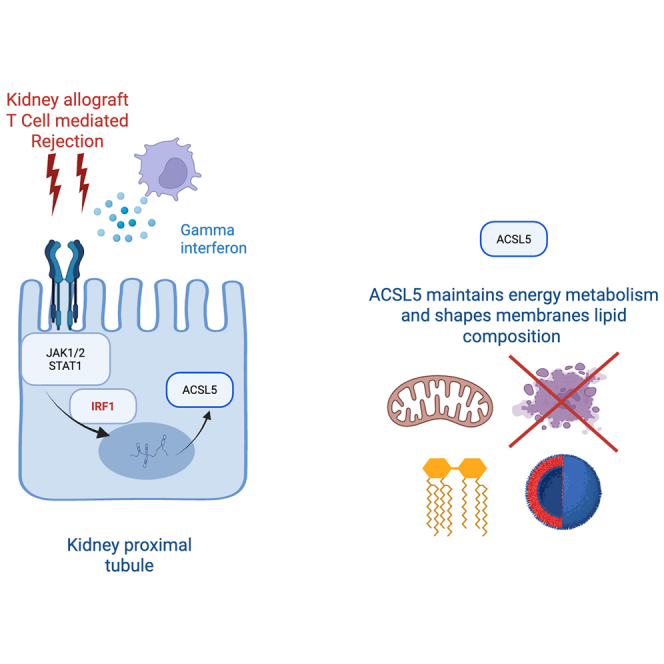

## Introduction

A distinctive feature of kidney injury is the alteration in lipid metabolism in kidney tubular cells. This process impairs cellular signaling, induces an inflammatory/fibrogenic phenotype, and activates regulated cell death.^1^^,^^2^^,^^3^^,^^4^ The availability and metabolism of fatty acids (FA) are of critical importance in maintaining cellular homeostasis within the tubule.^5^ FAs are stored as triacylglycerols (triglycerides, TG) in lipid droplets, acting as a reserve of energy. Furthermore, FAs are constituents of glycerophospholipids (phospholipids), which form cell membranes and act as intracellular mediators. Any disruption to the metabolic flux of FAs within the cell will have an impact on the cell’s energy metabolism or the composition of cell membranes, with critical consequences on kidney cells and tissue integrity. To illustrate, the accumulation of free FA, ceramides (Cer), and diacylglycerols (DG) promotes lipotoxic cellular malfunction in renal cells associated with organelle damage, disruption of intracellular signaling pathways, release of pro-inflammatory and pro-fibrotic factors, and apoptosis.^6^^,^^7^^,^^8^ Furthermore, defective oxidation of FA (FAO) in the kidney tubule, a feature of ischemic kidney injury, is a key driver of the transition and progression to chronic kidney disease.^9^^,^^10^ Finally, the specific FA composition of cell membranes is a determining factor in the susceptibility to ferroptosis, a regulated cell death pathway that occur in the kidney and is associated with iron-dependent dysregulation of phospholipid peroxidative activity.^11^^,^^12^^,^^13^

FA must be activated by acyl-CoA synthases in order to be further metabolized.^14^ The long-chain acyl-CoA synthetase (ACSL) family includes five different ACSL isoforms (ACSL1, 3, 4, 5, and 6), which are located on the endoplasmic reticulum (ER), mitochondrial outer membrane, cytosol, or plasma membranes.^15^ ACSL2 is no longer classified as it actually corresponds to ACSL1.^16^ ACSL isoforms catalyze FA with chain lengths from 12 to 20 carbon atoms to form acyl-CoA in the cytoplasm for lipogenesis and FAO. The role of ACSL activity in kidney pathophysiology remains unclear, with the exception of the emerging role of ACSL4 in acute and chronic models of kidney injuries.^12^^,^^17^^,^^18^^,^^19^^,^^20^^,^^21^ Indeed, ACSL4 activates long-chain polyunsaturated FA (PUFA) to PUFA-CoA products^22^ and enriches cell membranes with PUFA-containing phospholipid species that can be oxidized and accelerate cell death by ferroptosis.^12^^,^^13^^,^^23^ ACSL5 is located in the mitochondria and ER, and catalyzes the formation of acyl-CoAs using FA with 16 and 18 carbons as substrates (palmitate, stearate, with a preference for unsaturated FAs, such as palmitoleic, oleic and linoleic acids^24^).^15^^,^^25^^,^^26^ A renal FA-related gene signature comprising upregulation of *ACSL5* has recently been identified by our laboratory as being systematically associated with acute and chronic kidney injury,^27^ while another study showed it is downregulated during epithelial-mesenchymal transition.^28^ This suggests that ACSL5 activity may be involved in kidney pathophysiology, but its impact on kidney tubule health and its regulatory mechanisms remain unknown.

In this study, we integrated transcriptomic, metabolomic and lipidomic data from cohorts of kidney transplant recipients and cellular models, and molecular biology tools to demonstrate that *ACSL5* is a gene regulated by gamma interferon (IFNγ) and that it affects the energetic metabolism and lipidomic composition of kidney tubular cells. Furthermore, our findings indicate that *ACSL5* is overexpressed in human transplanted kidneys during rejection, and that it plays a distinct role in allograft response to injury, with prognostic implications.

## Results

### *ACSL5* expression is associated with cellular rejection in human kidney allografts

**Figure 1:**
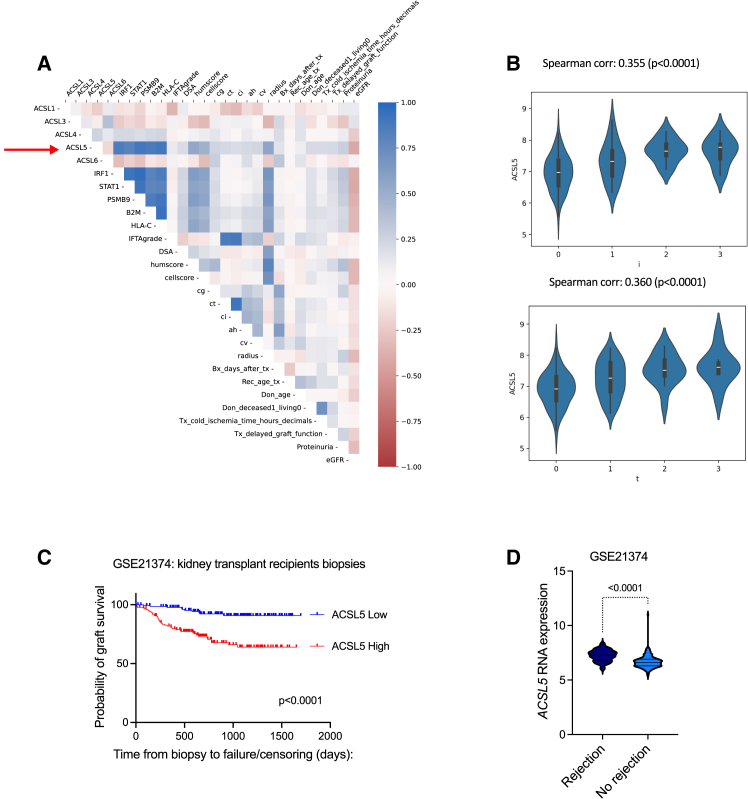
*ACSL5* is a molecular marker of rejection in human kidney allografts (A) Overall correlation heatmap between genes transcripts, clinical, functional and histological parameters using Pearson r scores in 224 protocol kidney allograft biopsy samples from 224 single kidney allograft recipients (protocol and for cause biopsies). Expression of transcripts was measured by microarray analysis of mRNA. *ACSLs* (Acyl-CoA synthetase long chain members), *IRF1* (interferon regulated gene 1), *STAT1* (signal transducer and activator of transcription 1), *PSMB9* (proteasome 20S subunit beta 9), *B2M* (beta 2 microglobulin), HLA-C (human leucocyte antigen C). DSA, donor specific antibody; Banff scores abbreviations: IFTA, interstitial fibrosis-tubular atrophy; cg, chronic glomerulitis; ct, tubular atrophy; ci, interstitial fibrosis; ah, arteriolar hyalinosis; g, glomerulitis; ptc, peritubular capillaritis; v, endarteritis; i, interstitial inflammation; t, tubulitis; humscore (humoral histologic score): g+ptc+localC4d+v+cg; cellscore (cellular histologic score): i+t+v; Radius (Activity index): (0.049∗t + 0.061∗i + 0.066∗v + 0.062∗g + 0.062∗ptcitis + 0.050∗thrombi +0.052∗C4D_ptc + 0.169∗DSA). (B) Expression of *ACSL5* according to the severity of interstitial inflammation (I score) and tubulitis (T score) according to the Banff classification. Data are represented as violin plots showing the median and quartiles. (C) Kaplan-Meier curves for the association between *ACSL5* transcripts levels and post biopsy graft survival in the cohort of 282 kidney transplant recipients who had a clinically indicated biopsy, according to the median of *ACSL5* transcripts distribution. Survival rates were compared with a Log Rank test. The microarray gene expression data are available at the Gene Expression Omnibus database under the accession number GSE21374. (D) *ACSL5* transcripts levels according to the histological diagnosis of rejection in the cohort of 282 kidney transplant recipients who had a clinically indicated biopsy. Data are represented as violin plots showing the median and quartiles. *p* values were computed with an unpaired t test.

We first sought to investigate the relevance of ACSLs isoforms expression in the human transplanted kidney. We conducted a comprehensive analysis of a multicenter cohort to gain a deeper understanding of the clinical and histologic factors associated with *ACSL*1, 3, 4, 5, and 6 expressions in kidney transplant biopsies.^29^ We performed an integrated analysis of allograft microarray data with extensive phenotypic characterization of 224 kidney transplant recipients over the course of six years as detailed elsewhere.^29^ Among the members of the *ACSL* family, *ACSL5* displayed the strongest anticorrelation with graft function, and was the only isoform positively correlated with markers of rejection and immune activity in the Banff classification (Figure 1A). In addition, the level of expression of the gene was proportional to with the severity of the rejection, as assessed by tubulitis and inflammation scores (Figure 1B).

To confirm the clinical significance of this discovery, we analyzed data from public repositories on the transcriptome of mRNA extracted from biopsy samples of 282 human kidney transplants (GEO database accession n^o^. GSE21374). This allowed us to assess the correlation between *ACSL5* and subsequent graft loss.^30^ The expression levels of the gene were categorized according to the median value of the distribution, resulting in the formation of two groups: high and low expression levels. The *ACSL5* high-expression level group had a significantly higher proportion of patients that progressed to graft loss after biopsy compared to the low-expression level group (log rank test, *p* < 0.0001) (Figure 1C). In addition, *ACSL5* levels were significantly higher in biopsies with features of rejection in this cohort (Figure 1D). When multivariate regression was performed with a Cox model that included the presence of rejection on the biopsy, *ACSL5* expression was found to have an independent prognostic value on graft survival (Table 1).

**Table 1:** Determinants of the graft transplant outcome at the time of biopsy (Cox proportional hazards model)

Hazard ratios	Variable	Estimate	95% CI (profile likelihood)
exp(β1)	ACSL5 Median [High]	4,578	2,293 to 9,948
exp(β2)	Rejection/non rejection:[Rej]	1,123	0,6197 to 2,012

Time variable: time from biopsy to failure/censoring (days; censor/event variable failed = 1/non failed; regression type: Cox regression; estimation method: exact.

### The composition of urine fatty acids is affected by kidney allograft rejection

**Figure 2:**
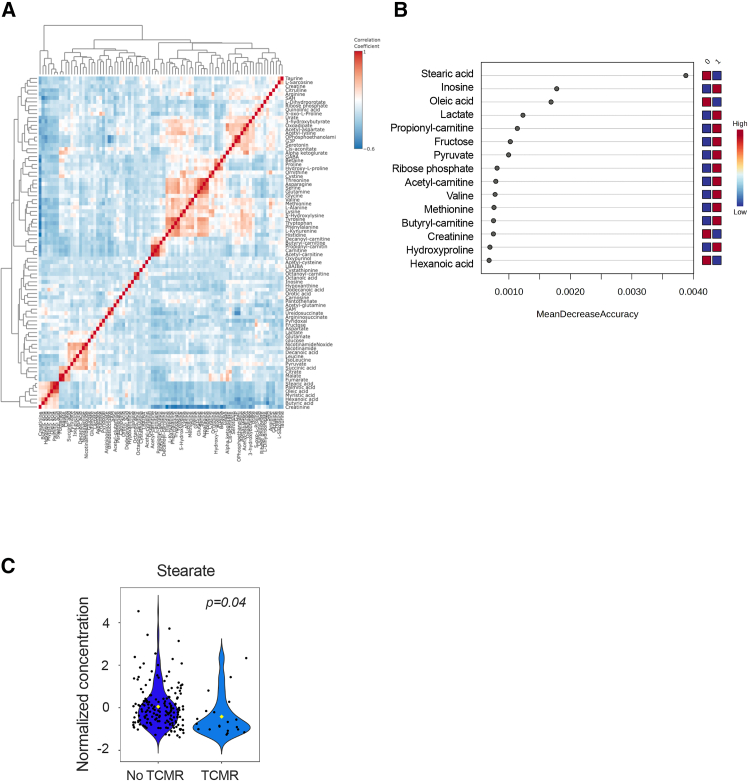
The composition of urine FA is affected by kidney allograft rejection (A) Overall correlation heatmap between metabolites using Pearson r scores in the 193 KTR urine samples collected 3 months after kidney transplantation and who underwent of protocol biopsy. (B) Significant metabolites in the 193 KTR urine samples collected 3 months after kidney transplantation and who underwent of protocol biopsy identified by random forest classification between group A and group B. Metabolite importance (top 15) are calculated by mean decrease accuracy for classification between group 0 (no inflammation) and group 1 (presence of inflammation, I and/or T > 1). ntree = 500, OOB error rate = 0.166, class error rate: group 0 = 0.00617 and group 1 = 1. The features are ranked by the mean decrease in classification accuracy after permutation. Dark red indicates that a feature (a metabolite) is enriched in a group. (C) Box and whiskers plots representing the distribution of stearate urine levels according to the presence of inflammation in the kidney allograft of the 193 KTR urine samples collected 3 months after kidney transplantation. *p* values were computed with an unpaired t test.

Having shown that *ACSL5* expression is associated with kidney transplant rejection, we tried to find out if there was a corresponding urinary metabolomic signature. For this, we conducted a targeted metabolite profiling of urines samples from 193 kidney transplant recipients collected three months after transplantation at the time of a protocol biopsy. This was done using liquid chromatography coupled to tandem mass spectrometry with SeQuant ZIC-pHilic columns, which permit the detection of complex hydrophilic and polar compounds.^31^ The clinical characteristics of the study population are presented in . The correlation analysis of metabolite levels revealed a clustered variability of molecules involved in carnitine-FA esters, tricarboxylic acid cycle intermediates, amino acids, and free FA with 16 and 18 carbons (palmitic acid, stearic acid, and oleic acid) (Figure 2A). We compared the urine metabolome of KTR according to the presence of T cell-mediated rejection according the Banff classification (*n* = 20).^32^ A urinary metabolic signature could not be identified by principal-component analysis (). However, a random forest model revealed that among the most significant predictor of the groups with and without rejection was the ACSL5 substrate stearate, and, to a lesser extent, oleate,^24^ which were lower in the urine of patients with rejection, compared to patients without (Figures 2B and 2C). Although the differences in FA contents are slight, which may be due to a lack of power related to the size of the cohort, these results suggest that the occurrence of kidney rejection is associated with changes in urinary FA composition, which may reflect the levels of ACSL5 substrates.

### *ACSL5* is an interferon γ-responsive gene in the tubule epithelium

**Figure 3:**
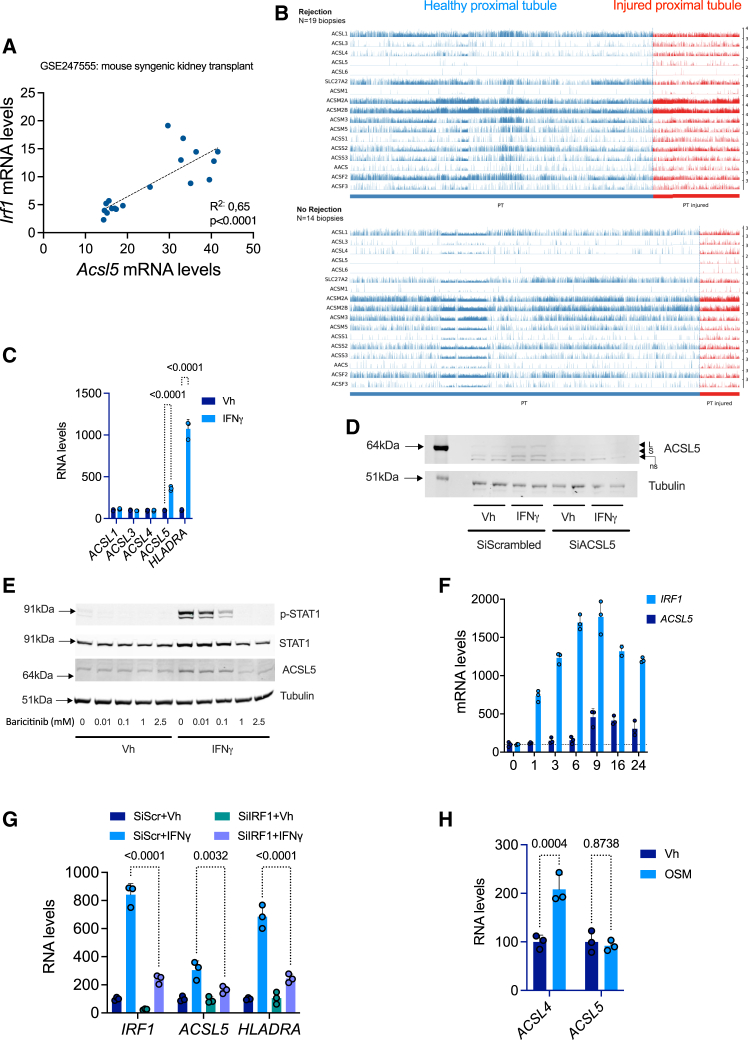
*ACSL5* is an interferon g-responsive gene (A) Simple linear regression between *Acsl5* and *Irf1* transcripts levels in mouse kidney isografts harvested at 24 h, 7 days and 14 days for RNA-Seq. Contralateral kidneys of donor mice were used as sham control. The RNAseq data are available at the Gene Expression Omnibus database under the accession number GSE144662. (B) Single cell analysis of a compiled scRNAseq dataset of 33 kidney transplant biopsies consisting of 16 biospies from 14 kidney transplant recipients followed in the University Hospitals Leuven, Belgium^34^ and 17 biopsies obtained from various publicly available scRNA-seq datasets (GSE140989,^35^GSE145927^36^ and PRJNA974568^37^), encompassing both allograft rejection and non-rejection biopsies. the analysis was restricted to the proximal tubules and to the gene involved in the activation of fatty acids. (C) *ACSL1*, *3*, *4*, *5*, and *HLADR* transcripts levels measured by RT-qPCR in HK-2 cells incubated with 100 ng/mL IFNg or vehicle (Vh, water). Data are represented as histogramms showing the mean and standard deviation. *p* values were calculated with a two-way ANOVA followed by a Šídák’s multiple comparisons test. *n* = 3 replicates per condition. (D) ACSL5 and tubulin protein levels in HK-2 cells transfected with siRNA directed against *ACLS5* RNA (siACSL5) or with control siRNA (siScrambled) and incubated with 100 ng/mL IFNg or vehicle (Vh, water) for 24 h. The immunoblot shown is representative of 3 independent experiments. L denotes the long isoform, s the short isoform, ns is a no-specific band. (E) phosphoSTAT1, STAT1, ACSL5, and tubulin protein levels in HK-2 cells exposed to increasing concentrations of baracitinib and incubated with 100 ng/mL IFNg or vehicle (Vh, water) for 24 h. The immunoblot shown is representative of 2 independent experiments. (F) Time course expression of *IRF1* and *ACSL5* transcripts measured by RT-qPCR in HK-2 cells incubated with 100 ng/mL IFNg or vehicle (Vh, water) for 24 h (*n* = 3 replicates per condition). Data are represented as histogramms showing the mean and standard deviation. *p* values were computed with a one-way ANOVA followed by a Dunnett’s multiple comparisons test. (G) *IRF1*, *ACSL5*, and *HLADRA* transcripts measured by RT-qPCR in HK-2 cells transfected with siRNA directed against *IRF1* RNA (siIRF1) or with control siRNA (siScr) incubated with 100 ng/mL IFNg or vehicle (Vh, water) for 24 h (*n* = 3 replicates per condition). Data are represented as histogramms showing the mean and standard deviation. *p* values were computed with a two-way ANOVA followed by a Tukey’s multiple comparisons test. (H) *ACSL4 and ACSL5* transcripts levels measured by RT-qPCR in HK-2 cells incubated with 20 ng/mL OSM for 24 h (*n* = 3 replicates per condition). Data are represented as histogramms showing the mean and standard deviation. *p* values were computed with a two-way ANOVA followed by a Šídák’s multiple comparisons test.

IFNγ signaling is a hallmark of the alloimmune activity in the transplanted kidney, to such an extent that it has been proposed as a molecular surrogate for rejection of graft biopsies.^33^ We analyzed the coexpression of a panel of IFNγ-responsive genes such as interferon regulated factor 1 (*IRF1*), signal transducer and activator of transcription 1 (*STAT1*), proteasome 20S subunit beta 9 (*PSMB9*), beta 2 microglobulin (*B2M*) and human leukocyte antigen C (*HLAC*) in the kidney transcriptomic cohort and we found that among ACSL isoforms, only *ACSL5* expression was positively correlated with all these genes (Figure 1A). Interestingly, the analysis of bulk RNA-sequencing data at different time points on a murine syngenic kidney transplantation model showed that *Irf1* and *Acsl5* were highly correlated at each time points (Figure 3A).

Our findings suggest that *ACSL5* may be an IFNγ-responsive gene. To substantiate this hypothesis, we conducted experiments to test if *ACSL5* is expressed in response to IFNγ in the tubular epithelium. In order to ascertain if the kidney tubule express *ACSL5* during rejection in the kidney transplant, an analysis was conducted of single-cell RNA sequencing (scRNAseq) data obtained from kidney transplant biopsies that were normal or diagnosed with rejection. The compiled scRNA-seq dataset included 86,845 individual cells from 33 biopsies. In the subset of 19 rejection biopsies, we identified 15,074 proximal tubule (PT) cells and 4,342 injured PT cells as defined by the expression of *HAVCR* and *Vimentin*. All tested genes showed significant differential expression between PT and injured PT cells (). *ACSL5* was rarely expressed in healthy PT cells (Figure 3B), leading to the highest fold change between PT and injured PT cells, with an average fold change of 7.0 (*p* < 0.001). On the subset of 14 No Rejection biopsies, 9,654 PT and 1,102 PT injured cells were identified. All genes tested, excepted *ACSM1*, showed significant differential expression between PT and injured PT cells (). *ACSL5* exhibited a similar upregulation in PT injured cells as observed on rejection biopsies. These data indicate that *ACSL5* is almost not expressed in the PT cells without sign of injury, but was significantly expressed in injured PT cells *in vivo*.

When the human renal epithelial cells (HREC) of proximal tubule origin (HK2 cells) were incubated with IFNγ, the expression of *ACSL5* transcripts was significantly increased, while the other *ACSL* isoforms remained unaltered, and the IFNγ-responsive gene *HLADRA* exhibited a marked elevation in expression (Figure 3C). At the protein level, IFNγ induced the expression of the 2 isoforms of ACSL5, which are due to presence of two in-frame AUG-translational initiators leading to a long isoform (739 amino acids) and a short isoform (683 amino acids)^25^ (Figure 3D). The specificity of the antibody (ab272556) is evidenced by the effective inhibition of the expression of both bands (but not a lower non-specific band) following incubation with small interfering RNAs (siRNAs) directed against *ACSL5* (Figure 3D). Furthermore, the expression of ACSL5 depends on STAT1 signaling since inhibition of JAK1 and STAT1 phosphorylation by baricitinib completely abolished the induction of ACSL5 expression upon exposure to IFNγ (Figure 3E). A time course analysis of *ACSL5* transcript levels following IFNγ induction demonstrated a significant upregulation of *ACSL5* observed between 6- and 9-h post-incubation. This suggests that ACSL5 is involved in a secondary wave of IFNγ-stimulated gene transcription, rather than in the primary response to IFNγ (Figure 3F). The *IRF1* gene expression is induced during the primary response to IFNγ via association of STAT1 homodimers and regulates the second wave of IFNγ-responsive genes.^38^ Critically, the 5′ region of the *ACSL5* gene is highly enriched for *IRF1* consensus binding sites, suggesting that this transcription factor regulates *ACSL5* expression (). Moreover, a time course analysis of transcript levels following IFNγ induction demonstrated a very early upregulation of *IRF1* observed after 1-h post-incubation, well before that of *ACSL5* (Figure 3F).

siRNA-mediated RNA interference against *IRF1* attenuated *ACSL5* expression upon IFNγ, as well as other IRF1-dependent genes such as *HLADRA* used as positive control (Figure 3G). We tried to use antibodies directed against IRF1 to validate the results by western blot. The only one of the three tested in the Human Protein Atlas (https://www.proteinatlas.org/ENSG00000125347-IRF1/summary/antibody) that seemed to work (albeit with spurious bands and inaccurate mass, Abcam Cat#ab26109, RRID:AB_775784) did not give any conclusive results in our hands. As evidence of the specificity of the STAT1-IRF1 pathway in regulating *ACSL5* expression, oncostatin M, a potent activator of STAT3 in the interleukin family 6 signaling pathways had no effect on *ACSL5* expression whereas it induced the expression of *ACSL4*^39^ (Figure 3H). Taken together, these results indicate that *ACSL5* is an IFNγ responsive gene in kidney tubule cells in culture under the control of IRF1 signaling.

### IFNγ-induced ACSL5 contributes to the maintenance of energy metabolism in the tubular epithelium

**Figure 4:**
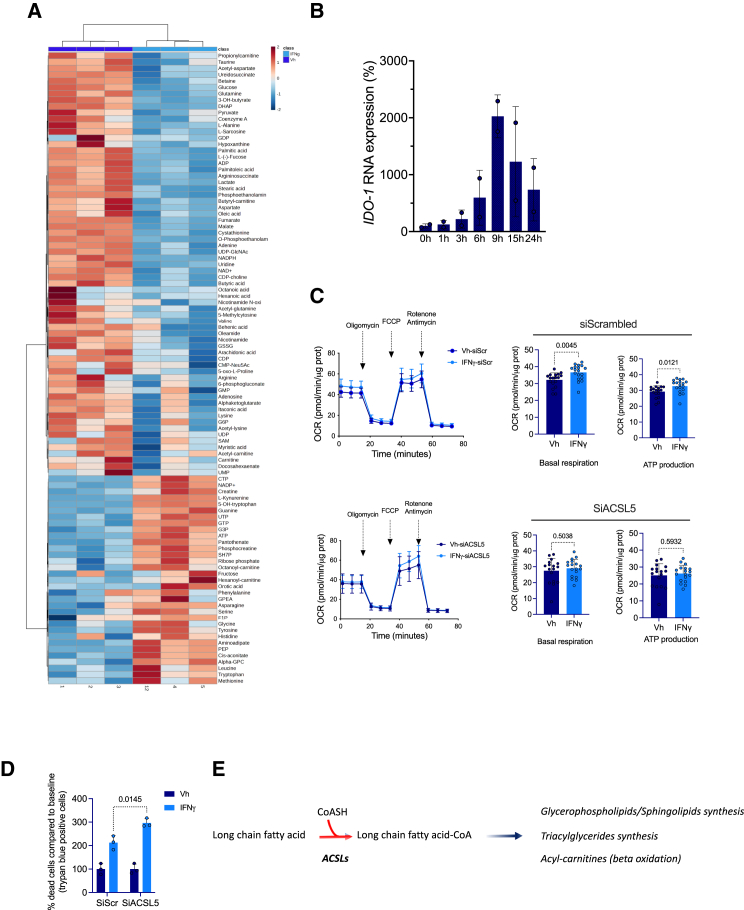
IFNγ-induced *ACSL5* sustains energy metabolism in the tubular epithelium (A) Heatmap showing hierarchical clustering using Ward’s algorithm for the 100 most significantly regulated metabolites (by ANOVA) in of HK2 cells incubated with 100 ng/mL IFNγ or vehicle (Vh, water) for 24 h (3 replicates). (B) Time course expression of *ID O -1* transcripts measured by RT-qPCR in HK-2 cells incubated 100 ng/mL IFNγ or vehicle (Vh, water) for 24 h (*n* = 2 replicates per condition). Data are represented as histogramms showing the mean and standard deviation. (C) Oxygen consumption rate (OCR) measured by SeaHorse Bioanalyzer in HK-2 cells transfected with siRNA targeting *ACSL5* under basal conditions and in response to incubation with 100 ng/mL IFNγ or vehicle (Vh, water) for 24 h. Arrows indicate the time of the addition of each reagent. Quantification of basal respiration and ATP production are shown on the graphs (right panels). Data are represented as histogramms showing the mean and standard deviation. *p* values were calculated with an unpaired t test (*n* = 18). (D) Trypan blue staining of HK-2 cells transfected with siRNA directed against *ACLS5* RNA (siACSL5) or with control siRNA (siScrambled) and incubated with 100 ng/mL IFNγ or vehicle (Vh, water) for 24 h (*n* = 3 replicates per condition). Data are represented as histogramms showing the mean and standard deviation. *p* values were computed with two-way ANOVA followed by a Šídák’s multiple comparisons test. (E) Metabolism of FA by ACSLs, and fate of activated fatty acids.

We next sought to ascertain the influence of the IFNγ-*ACSL5* signaling on cellular energy metabolism. Given the multifaceted impact of IFNγ on cellular metabolism,^40^^,^^41^^,^^42^ we conducted an incubation experiment with HREC cells, followed by a metabolomic analysis of the cell lysates. This analysis was conducted under the same analytical conditions as those employed for urine samples. We first performed hierarchical clustering of the 100 molecular species with the highest distribution variance between the two conditions to reveal compositional changes in response to IFNγ. This showed that IFNγ profoundly altered the intracellular metabolic profile of HK-2 cells. (Figure 4A). Among the metabolites enriched with IFNγ compared with the vehicle condition, we found as expected L-kynurenine, a product of indoleamine 2,3-dioxygenase (IDO1), an enzyme that provides the limiting step in kynurenine biosynthesis by catabolism of tryptophan and which is highly responsive to IFNγ in HREC (Figure 4B). Under IFNγ, the energy metabolism was clearly impacted with an increase in ATP content, a decrease in carnitines derivates, and a decrease in TCA cycle intermediates (alpha-ketoglutarate, fumarate, malate). IFNγ also impacted the Kennedy pathway of phosphatidylcholine (PC) and phosphatidylethanolamine (PE) synthesis (decreased levels of cytidine diphosphate-choline, O-phosphoethanolamine and ethanolamine phosphate and increases in glycerophosphorylcholine). The third main metabolic pathway impacted by IFNγ appeared to be the pyrimidine synthesis pathways, with significant reduction in intracellular levels of pyrimidine nucleotides precursors, such as cytosine diphosphate, uridine, uridine mono- and di-phosphate, and increases in uridine tri-phosphate (Figure 4A). In line with the FA urine profile associated with rejection, we observed a reduction in the cellular contents of ACSL5 substrates stearic acid, oleic acid, and also palmitoleic acid and coenzyme A.

This metabolomic profiling indicates that IFNγ affects cellular energy metabolism. To ascertain the functional impact of IFNγ on cellular energy metabolism, we examined the energetic profile of HREC exposed to IFNγ using the Seahorse Bioanalyzer. We measured the oxygen consumption rate (OCR) of HK-2 cells incubated with IFNγ and found that basal respiration and ATP production were higher when IFNγ was added to cells (Figure 4C). When *ACSL5* was knocked-down by siRNA-mediated RNA interference, the stimulatory effect of IFNγ in OCR was lost. Critically, the reduction of ACSL5 activity by RNA interference upon IFNγ exposure significantly increased cell death, as evaluated by trypan blue inclusion (Figure 4D). These results indicates that ACSL5 activity is involved in tubular energy metabolism and oxidative phosphorylation in cells exposed to IFNγ, and that these properties likely play a substantial role in keeping cell viability. The exact mechanisms that are at play in this system have yet to be determined. Contents in FA species with 16 and 18 carbons, as well as coenzyme A, were reduced in HREC exposed to IFNγ, which may be a consequence of an increased metabolism (Figure 4E).

### ACSL5 shapes the tubule cell lipidome in response to IFNγ

**Figure 5:**
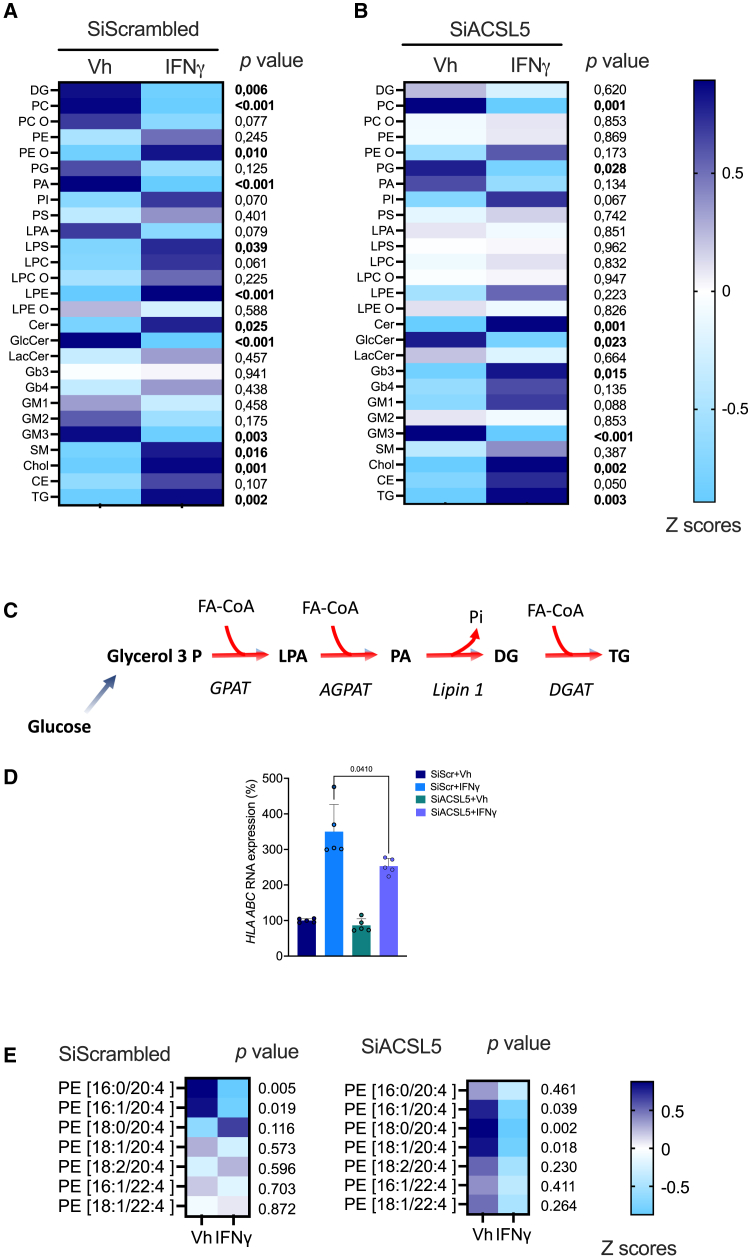
ACSL5 shapes the tubule cell lipidome in response to INFγ (A and B) Heatmap showing the relative composition (mean *Z* score) in lipid classes identified in HK-2 cells after transfection with siRNA directed with control siRNA (siScr) (A) or against *ACLS5* RNA (siACSL5) (B) and incubated with 100 ng/mL IFNγ or vehicle (Vh, water) for 48 h (*n* = 3). Each line corresponds to the proportional composition in lipid class after autoscaling (i.e., normalizing the data by centering the mean and dividing by the standard deviation of each variable). (C) Triglycerides synthesis pathway. GPAT: glycerol-3-phosphate acyl transferase, AGPAT, 1-acylglycerol-3-phosphate-*O*-acyltransferase; DGAT, diacylglycerol O-acyltransferase; LPA, lysophosphatic acid; PA, phosphatic acid; DG, diacylglycerol; TG, triacylglycerol. (D) *HLAABC* transcripts measured by RT-qPCR in HK-2 cells transfected with siRNA directed against *siACSL5* RNA (siIRF1) or with control siRNA (siScr) incubated with 100 ng/mL IFNγ or vehicle (Vh, water) for 24 h (*n* = 3 replicates per condition). Data are represented as histogramms showing the mean and standard deviation. *p* values were computed with a two-way ANOVA followed by a Tukey’s multiple comparisons test. (E and F) Heatmap showing the relative composition (mean *Z* score) in C20–4 and C22-4-PE identified in HK-2 cells after transfection with control siRNA (siScr) (A) or with siRNA directed against *ACLS5* RNA (siACSL5) (B) and incubated with 100 ng/mL IFNγ or vehicle (Vh, water) for 48 h (*n* = 3). Each line corresponds to the proportional composition in lipid class after autoscaling (i.e., normalizing the data by centering the mean and dividing by the standard deviation of each variable).

ACSL5 plays a central role in the regulation of FAO activity and TG synthesis through the activation of 16- and 18-carbons FA,^43^^,^^44^ and our results highlight the importance of its role in the energy metabolism of kidney tubular cells during exposure to IFNγ. However, ACSL5 impacts the lipidomic landscape that extend beyond the field of glycerolipids (lipids formed by the condensation of 1, 2, or 3 FA molecules on glycerol),^45^ and may exert a more pervasive influence on cellular lipid composition in these circumstances. We therefore characterized the impact of ACSL5 on the cellular lipidome to gain a comprehensive understanding of the impact of IFNγ and ACSL5 on the lipid composition of kidney tubule cells. To this end, HREC were incubated for 48 h with or without IFNγ. Following this, the samples were subjected to lipidomic profiling by high resolution spectrometry, which identified more than 500 lipid species. Regulated lipid classes identified in the cells clearly differentiated IFNγ and vehicle samples (Figure 5A). One notable change was an increase of FA storage lipids consisting mainly in TG, and a reduction of TG synthesis intermediates, phosphatic acids (PA), DG, and in a lesser extent lysophosphatic acid (LPA, a phospholipid with a single FA), which is consistent with an activated metabolic flux directed toward TG synthesis (Figure 5C). With regard to phospholipids, a significant decrease was observed in the levels of PC and ether-linked PC (PC O) (Ether phospholipids have a similar structure to phospholipids, but differ in that they have at least one ether bond at carbon 1 of glycerol instead of an ester bond) whereas PE and ether-PE (PE O) were increased in cells incubated with IFNγ, corroborating our metabolomic data that suggested that the Kennedy pathway of PC and PE synthesis was affected by IFNγ. Finally, IFNγ affected sphingolipids composition with an increase in Cer contents and a decrease in glucosylceramides and gangliosides GM3.

We next investigated the effects of ACSL5 on cell lipidome. *ACSL5* knock-down had a substantial effect on the whole lipidome at baseline, as evidenced by the principal-component analysis (). Compared to conditions in which scrambled siRNA was used, silencing of *ACSL5* in IFNγ treated cells was associated with a far less contrasted profile of expression of a number of glycerolipids. To illustrate, PE O, lysophosphatidylserine (LPS), lysophosphatidylcholine (LPC), and lysophosphatidylethanolamine (LPE), which exhibited an increase under IFNγ, no longer demonstrated this trend when *ACSL5* was rendered invalid. Conversely, lipid classes, such as DG, PC, PC O, PA, and LPA, whose contents were reduced in the IFNγ incubation condition, were no longer affected when *ACSL5* was invalidated (Figure 5B), suggesting that ACSL5 is a driver of the effects of IFNγ on glycerolipid composition. On the other hand, we observed a number of sphingolipids for which the ablation of *ACSL5* led to increased levels upon IFNγ. This effect was particularly noticeable for globotriaosylceramide (Gb3 globoside, one of the targets of Shiga toxin). *ACSL5* invalidation also enhanced the accumulation of Cer contents induced by IFNγ, without a distinct compositional pattern of molecular species being observed (). These results show that ACSL5 silencing has a marked effect on the lipid composition of cells in the basal state, which is consistent with the central role of this enzyme in FA metabolism, but more importantly, ACSL5 plays an important role in the membrane lipidomic reprogramming that occurs during exposure to INFγ, since under conditions where ACSL5 is silenced, the effect of INFγ on lipid remodeling is significantly reduced compared to cells expressing ACSL5.

It has recently been showed that ACSL5-activated FA metabolism promotes MHC-I antigen presentation trough the activation of NLRC5, a transcription factor that may sense FA metabolism alteration.^46^ In line with this, in HREC incubated with IFNγ and invalidated for ACSL5, the expression of the MHC-I genes HLA-A, -B, and -C was reduced compared with the control condition, suggesting that ACSL5 activity may participate in antigen presentation under IFNγ exposure (Figure 5D).

Since IFNγ sensitizes cells to ferroptosis,^47^ we evaluated the composition in PE containing FA 20-4 (arachidonic acid) and FA 22-4 (adrenic acid), which are potent targets of peroxidation during ferroptosis.^12^ The inactivation of ACSL5 in Vh-treated cells appears to be associated with an increased relative expression of AA-containing PE. IFNγ reduced the contents levels for PE 16:0/20:4 and PE 16:1/20:4, but promoted a slight increase in PE 18:0/20:4, one of the prime target of peroxidation during ferroptosis with PE 18:1/20:4^12^ (Figure 5E). Critically, invalidating *ACSL5* led to a substantial reduction in contents of PE 18:0/20:4 and PE 18:1/20:4, suggesting that ACSL5 may participate in the enrichment of membranes in C20:4 PUFA (Figure 5E).

These findings illustrate that ACSL5 activity exerts a substantial impact on the lipid composition of HREC in response to IFNγ exposure. This is evidenced by its role in reducing the accumulation of PE, numerous lysophospholipids, as well as Cer. Furthermore, ACSL5 seems to play part in the enrichment of membranes PE 18:0/20:4 and PE 18:1/20:4, with are known ferroptosis targets.

## Discussion

We show that *ACSL5* is expressed by tubular epithelial cells in response to IFNγ signaling, and that its activity has significant implications for maintaining cellular energy metabolism and in reshaping the lipidomic composition of kidney tubular cells with broad biological implications. Moreover, we demonstrate that *ACSL5* is overexpressed in human transplanted kidneys during rejection, and that it appears to have a role of its own in allograft response to injury with a prognostic value. This is the demonstration that *ACSL5* is involved in the context of alloimmunity and may play a detrimental role in the survival of kidney transplants. This observation is particularly relevant to the recently demonstrated a beneficial role of *ACSL5* in cancer, where overexpression of *ACSL5* is beneficial in the context of antitumor immunity by enhancing tumor immunogenicity.^46^ This places *ACSL5* as a potential candidate biomarker and a promising therapeutic target, situated at the nexus of IFNγ signaling, anti-tumor immunity and alloimmunity.

ACSL5, which has a wide range of preference for the generation of FA-CoA, has been implicated in FA partitioning to TG synthesis. Nevertheless, there is a certain degree of debate in the scientific literature regarding the characteristics ascribed to it in the context of its role in the synthesis of TG or the production of activated FA, which serve as substrates for mitochondrial beta-oxidation.^14^^,^^43^^,^^48^^,^^49^ It is contended that these two metabolic functions are exclusive and contingent upon the specific models and experimental conditions employed. Our study indicates that ACSL5, in the presence of IFNγ, may participate in the activation of FA to fuels ATP production. These properties appear to be crucial for cellular survival, as evidenced by the increased cell death observed in the absence of ACSL5.

The latter data on cell viability *in vitro* may appear to be at odds with the findings of our translational observations in cohorts of kidney transplant patients. Indeed, our results indicate that *ACSL5* is overexpressed in transplanted kidneys in a manner contingent on that of other genes regulated by IFNγ and is associated with a histological phenotype of rejection. Furthermore, our multivariate analysis assigns *ACSL5* expression a negative prognostic value independent of that of the accompanying rejection. This means that this enzyme has a deleterious impact on kidney tissue homeostasis in response to IFNγ-induced stress and that the beneficial intrinsic cellular effects on energy metabolism do not add up to the full impact of ACSL5 activity on tubular cells, which may activate potentially harmful cell-extrinsic signals. In light of the complex biological ramifications of IFNγ-mediated signaling on cell biology and the profound impact of ACSL5 activity on the cellular lipidome, mechanistic hypotheses in line with alterations in biological functions contingent upon accessible lipid resources, such as intracellular vesicular transport and antigen presentation can be proposed.^50^^,^^51^ Indeed, the lipid composition of the cell, and in particular in FA, affects the capacity for major histocompatibility complex expression and antigenic presentation of cells.^52^^,^^53^ Critically, ACSL5 promotes antigen presentation of tumor cells, and sensitizes tumor to cytotoxic T lymphocytes-mediated cytotoxicity.^46^ Although partially unresolved, the mechanism is dependent on the presence of oleic acid isomers and the enzymatic activity of ACSL5. If a similar process occurs in kidney tubular cells in the context of alloimmunity, the increased expression of *ACSL5* in tubular cells during rejection could enhance cell immunogenicity and explains the poorer prognosis of grafts with high levels of *ACSL5*. This hypothesis remains to be tested.

Our lipidomic profiling demonstrates that *ACSL5* plays a role in the regulation of sphingolipid metabolism, and notably in limiting the accumulation of Cer, which have been observed to exert lipotoxicity in the kidney.^54^^,^^55^ The impact of ACSL5 activity in this context in the kidney tubule remains to be demonstrated but is probably important given the importance of Cer in senescence, apoptosis, and autophagy.^56^^,^^57^^,^^58^ ACSL5 has been shown to be involved in ceramide metabolism by forming a tri-enzyme complex composed of diacylglycerol O-acyltransferase 2 and ceramide synthase which transforms Cer to acylCer. This complex allows ceramide to be stored as an acylceramide in droplet lipids, thereby sequestering Cer in an inactive form and providing cytoprotection.^45^ In this model, inhibiting *ACSL5* leads to Cer accumulation, which is consistent with our findings.

In conclusion, we identified *ACSL5* a potential marker of kidney allograft rejection with a deleterious impact role on kidney transplant survival and an IFNγ-regulated gene that maintains energy metabolism in kidney tubular cells, and profoundly affect glycerolipid and Cer metabolism.

### Limitations of the study

In contrast to the findings of other studies, our analysis did not identify a distinct urinary metabolomic signature associated with cellular rejection in the cohort under investigation.^59^^,^^60^^,^^61^^,^^62^^,^^63^ The discrepancy may be attributed to various factors, including the analytical platform employed, the panel of metabolites that can be analyzed, the study design, the size of the cohort, the age of the samples, and other considerations such as the bioinformatic pipeline. These findings reiterate the challenge of obtaining urinary metabolomic results that are consistently reproducible across different cohorts and platforms. Notwithstanding this limitation, our study yielded a significant finding: the ACSL5 substrates contents were diminished in the urine of patients with kidney allograft rejection at three months. This observation aligns with the hypothesis that accelerated metabolism of these substrates may be linked to increased ACSL5 activity.

Another limitation of our study is that a more comprehensive insight into the function of ACSL5 in response to IFNγ in the transplanted kidney could be gained by utilizing a mouse model with *ACSL5* deficiency in the tubule and undergoing allogeneic kidney transplantation. However, this currently presents a significant technical challenge.

## Resource availability

### Lead contact

Further information and requests for resources and reagents should be directed to and will be fulfilled by the lead contact, Nicolas Pallet: Nicolas.pallet@aphp.fr.

### Materials availability

This study did not generate new unique reagents.

### Data and code availability

Expression profiling by RNA microarrays for the 224 human kidney transplant biopsies are available at GEO (GSE147089).

Metabolomic data are available as Mendeley Data, https://doi.org/10.17632/jcd93vd3d4.1. All are publicly available as of the date of publication.

The lipidomic standard reporting checklist (https://lipidomicstandards.org/) is available at the https://doi.org/10.5281/zenodo.10908592, and Lipidomic raw data are available as Mendeley data, https://doi.org/10.17632/hpht5tv5xz.1. All are publicly available as of the date of publication.

This paper does not report original code.

Any additional information required to reanalyze the data reported in this paper is available from the lead contact upon request.

## Acknowledgments

This work was funded by grants from l’Agence de la Biomédecine (Appel d’offre Recherche et Greffe).

## Author contributions

V.P. and A.P. performed experiments; J.L.S. performed lipidomic analysis; I.N. performed metabolomic analysis; M.N. and T.V. performed transcriptomic and single cell data mining; D.A. generated Necker’s kidney transplant recipients’ datasets and urine biobank. N.P. conceived, designed the project, analyzed dataset, and wrote the manuscript.

## Declaration of interests

The authors have no conflict of interest to disclose.

## STAR★Methods

### Key resources table

**Table:** 

REAGENT or RESOURCE	SOURCE	IDENTIFIER
**Antibodies**		

Rabbit Anti-ACSL5	Abcam	Cat # ab272556
Rabbit phospho Y701 STAT1	Abcam	Cat # ab109467
Rabbit monoclonal STAT1	Abcam	Cat # ab92506
Mouse Tubulin	Millipore Sigma	Cat# T9026

**Chemicals, peptides, and recombinant proteins**		

Recombinant Human IFN-gamma Protein	R&D System	Catalog #: 285-IF
Baricitinib	R&D System	Cat. No. 7222
Human OSM (rhOncostatin M)	R&D System	Cat# 295-OM/CF

**Critical commercial assays**		

Thermo Scientific Pierce BCA Protein Assay Kits	Thermo Fisher Scientific	Cat# 10741395
RNeasy Mini Kit	QIAGEN	Cat# 74106
Hight Capacity cDNA Reverse Transcription Kit	Applied Biosytem	Cat# 4368814
AB Solute Blue qPCR SYBR Green ROX Mix	ThermoScientific	Cat# AB-4162/B
Mycoalert Mycoplasma Detection Kit	Lonza	Cat# LT07-318

**Deposited data**		

Metabolomic data are available as Mendeley Data	This paper	https://doi.org/10.17632/jcd93vd3d4.1
The lipidomic standard reporting checklist (https://lipidomicstandards.org/) is available at the	This paper	https://doi.org/10.5281/zenodo.10908592
Lipidomic raw data are available as Mendeley Data,	This paper	https://doi.org/10.17632/hpht5tv5xz.1

**Experimental models: Cell lines**		

HK-2 Cell line	ATCC/LGS	ATCC-CRL-2190

**Oligonucleotides**		

AllStars Negative Control siRNA	QIAGEN	SI03650318
Hs_ACSL5_6	QIAGEN	SI04147738
Hs_ACSL5_7	QIAGEN	SI04170887
Hs_ACSL5_8	QIAGEN	SI04254530
Hs_ACSL5_9	QIAGEN	SI04256861
Hs_STAT1_7	QIAGEN	SI02662884
Hs_STAT1_6	QIAGEN	SI02662324
Hs_STAT1_12	QIAGEN	SI04950960
Hs_STAT1_9	QIAGEN	SI03119025
Hs_IRF1_5	QIAGEN	SI02628080
Hs_IRF1_4	QIAGEN	SI00034104
Hs_IRF1_2	QIAGEN	SI00034090
Hs_IRF1_1	QIAGEN	SI00034083
***Human Acsl1***: 5′ - CTCCAGTCCCCTGTGGTTTCT -3′; 5′ - GATCTGCCCTCCCGCTTACT -3′	This paper	N/A
***Human Acsl3***: 5′ - TTGACACAAGGGCGCATATC -3′; 5′ – CAGCTTCTGAGGGTGGCAAA - 3′	This paper	N/A
***Human Acsl4***: 5′ - TTCTCATTCTTCCCAACTTGGCT - 3′; 5′ - GGCAGATTGGGGGTGCAAAT - 3′	This paper	N/A
***Human Acsl5***: 5′ - TCCCCGAGCACGTTAGAAAG - 3′; 5′ - TTCTTCCTGCCACACGAGTC - 3′	This paper	N/A
***Human Ddit3***: 5′ - AGCCCTGTGGAACTGAGAGA - 3′; 5′ - CAGGTTTTCCATTTCGAAGC - 3′	This paper	N/A
***Human HspA5***: 5′ - CCTACGACGGCAAGGATTAC- 3′; 5′ - TGCCAGGTCAGTGTGA TCTC- 3′	This paper	N/A
***Human Il-6***: 5′ - GTGAAAGCTCTGGTCTCCCT - 3′; 5′ – TCA GTG CCT CCA GTT CCT TT 3′ - 3′	This paper	N/A
***Human Rpl13a***: 5′ - CCTGGAGGAGAAGAGGAAAGAGA - 3′; 5′ - GAGGACCTCTGTGTATTTGTCAA - 3′	This paper	N/A

**Software and algorithms**		

GraphPad Prism 10 for Mac	GraphPad Software	Version 10.0.2
ImageJ	https://imagej.nih.gov/ij/	Version 1.53.t

**Other**		

QuantStudio 7 Flex Real-Time PCR System	Applied Biosytems	N/A
Tecan Safire r plate reader	Tecan	N/A

### Experimental model and study participant details

#### Kidney transplant cohort for transcriptomic analysis of kidney biopsy

A total of 224 kidney allograft biopsy samples from 224 single kidney allograft recipients were collected in four European transplant centers between June 2011 and March 2017 in the context of the BIOMArkers of renal Graft INjuries (BIOMARGIN) study (www.biomargin.eu; clinicaltrials.gov number NCT02832661), and the Reclassification using OmiCs integration in KidnEy Transplantation (ROCKET) study. In these four clinical centers, protocol biopsies were performed at 3, 12, and sometimes at 24 months after transplantation in addition to the indication biopsies. Institutional review boards and national regulatory agencies (when required) approved the study protocol at each clinical center. The transcriptome of the kidney allograft biopsies specimens has been analyzed on Affymetrix GeneChips. The cohort is detailed in ^29^. The microarray gene expression data are available at the Gene Expression Omnibus database under the accession number GSE147089.

#### Necker kidney transplant recipient’s cohort for urinary metabolome

All consecutive patients (*n* = 405) who received a kidney transplant at our center between January 2010 and June 2012 were considered for this prospective, longitudinal, single-center cohort study. The reasons for exclusion were as follows: non-inclusion criteria (*n* = 48), primary non-function/early graft loss (*n* = 12), other study with urine monitoring (*n* = 16), patient death within the first 6 months (*n* = 7), and early loss of follow-up (*n* = 22). Three months following transplantation, urine samples were collected from 272 of the 300 individuals initially included in the study. Urine samples were deemed suitable for metabolome analysis in 248 kidney transplant recipients. Of this number of kidney transplant recipients, 193 were deemed to have an interpretable and adequate protocol biopsy. This study was approved by the Ethics Committee of Ile-de-France XI (#13016), and all of the participating patients provided written informed consent. The urine samples were subjected to centrifugation at 1000*g* for a period of 10 min, within a time frame of 4 h from the point of collection. The resulting supernatant was subsequently collected after centrifugation and stored at −80°C with protease inhibitors. The urine samples used in this study have been previously analyzed and published.^64^

#### Sex, gender, or both have no influence on the results of the study

##### Cell lines

HK2 cells (ATCC/LGC Standards (lot number 60352186) were established by transduction with human papilloma virus (HPV 16) E6/E7 genes from a primary proximal tubule cells culture from normal male adult human kidney cortex. Cell lines were authenticated by ATCC.

### Method details

#### Single cell analysis

Single cell analysis was performed on a compiled scRNAseq dataset of 33 kidney transplant biopsies consisting of 16 biospies from 14 renal transplant recipients followed in the University Hospitals Leuven, Belgium^34^ and 17 biopsies obtained from various publicly available scRNASeq datasets (GSE140989,^35^ GSE145927 ^36^ and PRJNA974568^37^), encompassing both allograft rejection and non-rejection biopsies. The detailed pre-processing methodology is available in Van Loon et al.^34^ Briefly, cells with fewer than 400 or more than 10,000 detected genes, as well as those containing more than 25% mitochondrial transcripts, were removed during preprocessing. Cell populations were annotated with canonical lineage markers, with injured PT cells identified by lesion markers *VCAM1* and *HAVCR1*. Log-transformed gene expression for individual cells was visualized using Scanpy’s tracksplot^65^ on raw, non-integrated data. Differential expression analysis was performed using Seurat V5’s FindMarkers^66^ function with MAST,^67^ incorporating a batch covariate to account for batch effects from the integration of multiple datasets. The differentially expressed genes analysis was restricted to the predefined set of ACSL and related genes.

#### Cells

HK-2 cells are cultured in Dulbecco’s Modified Eagle Medium (DMEM) containing 5 μg/mL insulin, 10 μg/mL human apotransferrin, 500 ng/mL hydrocortisone, 10 ng/mL epithelial growth factor, 6.5 ng/mL triiodothyronin, 5 ng/mL sodium selenite, 1% fetal calf serum, 25 IU/mL penicillin, 25 μg/mL streptomycin and 10 mM HEPES buffer. These cells lines are Mycoplasm free (Mycoalert Mycoplasma Detection Kit, Lonza).

#### RNA extraction and real-time quantitative polymerase chain reaction (RT-qPCR)

Total RNA was extracted using the RNeasy Mini Kit® (Qiagen) according to the manufacturer’s protocol. Transcript expression levels were quantified by SYBR green RT-qPCR using the QuantStudio™ 7 Flex Real-Time PCR System (Applied Biosystems). Vehicle-treated samples were used as controls, and fold changes for each gene tested were normalized to the ribosomal protein L13A (*RPL13A*) housekeeping gene. Relative expression levels were calculated using the 2^-ΔΔCT^ method.^68^ Primers sequences are listed in the key resources table.

#### Protein extraction and immunoblotting

Cells were washed in PBS and incubated for 15 min at 4°C and in NADOC (0.5% Tritonx-100, 0.5% sodium deoxycholate, 50 mM Tris, and 150 mM NaCl) lysis buffer (Thermo Fisher Scientific) with protease (HaltTM Protease Inhibitor Cocktail 100X, Thermo Fisher Scientific) and phosphatase inhibitors (HaltTM Phosphatase Inhibitor Cocktail 100X, Thermo Fisher Scientific). The extracts were centrifuged at 14,000 g for 20 min. Protein concentrations in the supernatant were measured using a Pierce BCA protein assay kit (Thermo Fisher Scientific) and a Tecan Safire® plate reader. Protein extracts (25 μg) were resolved by 4–12% SDS-PAGE (Invitrogen) and transferred to nitrocellulose membranes (iBlot, Invitrogen). Membranes were blocked with SEABLOCK blocking buffer (Thermo-Scientific) for 1 h at room temperature and then incubated overnight at room temperature with primary antibodies diluted in blocking buffer. The primary antibodies are listed in the key resources table. After washing in PBS-Tween buffer, the membranes were incubated with secondary antibodies conjugated to IRDye fluorophores. The infrared signal of the membranes was detected using an Odyssey detection system (Li-Cor biosciences).

#### siRNA transfections

Transient inactivation of *ACSL5* and *IRF1* genes were achieved using synthetic small interfering RNAs (siRNAs) designed and purchased from Qiagen and transfected using Lipofectamine 3000 (Invitrogen) according to the manufacturer’s protocol. Four different siRNA against the same target were transfected for ACSL5 and STAT1. Scrambled (control) siRNA have no homology to any known mammalian gene and was validated using Affymetrix GeneChip arrays and a variety of cell-based assays to ensure minimal non-specific effects on gene expression and phenotype. The list of siRNAs and their sequences is in the key resources table. Cells were incubated with siRNA for at least 24 h prior to experiments.

#### Cell death assay

##### Trypan blue exclusion

Cells were seeded in 6-well plates and treated with appropriate stimuli, and dead cells were counted at various times using the trypan blue dye exclusion assay. Medium containing floating cells was collected and centrifuged. Adherent cells were trypsinized and centrifuged. Pellets of floating and detached cells were resuspended in PBS, spread on Malassez cells, and counted with a phase contrast microscope. The percentage of cell death was determined by dividing the number of dead cells (stained blue) by the total number of cells and expressed as the percentage of dead cells under basal conditions. All values are the average of at least three independent experiments, each performed in triplicate or more.

#### Mitochondrial activity measurements

For oxygen consumption rate measurements using the Seahorse bioanalyzer, HK-2 cells were seeded at a density of 6 × 10^4^ cells per well in a collagen-coated XFe96 cell culture microplate. Twenty-four hours after plating, cells were incubated with siRNA targeting *ACSL5* or scrambled siRNA as negative control for 24 h. After 24 h, cells were incubated with IFNg for 24 h, and mitochondrial activity was assessed. Prior to measurement, cells were balanced for 1 h in unbuffered XF assay media (Agilent Technologies) supplemented for OCR analysis with 2 mM glutamine, 10 mM glucose, and 1 mM sodium pyruvate. For oxygen consumption rate (OCR) measurements, compounds were injected during the assay at the following final concentrations Oligomycin (ATP synthase inhibitor to measure respiration associated with cellular ATP production; 1 μM), FCCP (uncoupling agent to measure maximal respiratory capacity; 1 μM), Rotenone and Antimycin A (ETC inhibitors to measure non-mitochondrial respiration; 1 μM).

#### Targeted metabolomics

Metabolomics analyses were performed on cells in culture and urine sample. HK-2 cells were washed twice with ice-cold PBS, drained, snapped-frozen in liquid nitrogen and stored at −80°C until analyses. Urines were centrifugated 5 min at 4000 g and the supernatant was stored at −80°C until analyses. After addition of an extraction solution made of 50% methanol, 30% acetonitrile, and 20% water (1 mL/1.10^6^ cells or 500 μL for 20 μL urine), the samples were vortexed for 5 min at 4°C, and then centrifuged at 16,000 g for 15 min at 4°C.^31^ The supernatants were collected and separated by liquid chromatography-mass spectrometry using SeQuant ZIC-pHilic column (Millipore). The aqueous mobile-phase solvent was 20 mM ammonium carbonate plus 0.1% ammonium hydroxide solution and the organic mobile phase was acetonitrile. The metabolites were separated over a linear gradient from 80% organic to 80% aqueous for 15 min. The column temperature was 50°C and the flow rate was 200 μL/min. The metabolites were detected across a mass range of 75–1,000 m/z using the Q-Exactive Plus mass spectrometer at a resolution of 35,000 (at 200 m/z) with electrospray ionization and polarity switching mode. Lock masses were used to ensure mass accuracy below 5 ppm. The peak areas of different metabolites were determined using Thermo TraceFinder software using the exact mass of the singly charged ion and known retention time on the HPLC column. These metabolomics analyses are focused on small polar compounds in central carbon metabolism. We applied an established and largely referenced method for sample extraction and LC-MS analyses using pHILIC HPLC column for polar metabolites separation.^31^ Notably, we used the same extraction solution for cells and urine samples. As a part of the routine analytical pipeline, we apply the recommendations of the metabolomics Quality Assurance and quality Control Consortium (mQACC). The routine quality controls include regular equipment maintenance (Thermo), the use of standard operating procedures for sample extraction, storage and analyses. General practices also include weekly test runs to assure system stability and quality of runs. Regarding the QCs in relation to this study, we used (1) pooled interstudy QC, (2) process and extraction blanks, (3) system stability blanks, (4) solvents blanks, (5) long-term reference standard inter-laboratory QC mix to ensure system stability and (6) the samples were blinded and loaded in randomized order. The analyses of pooled samples QC showed no significant difference in metabolites levels between QCs. Data analysis was performed in the MetaboAnalyst 5.0 software.^69^ Feature filtering was based on Interquantile Range. Samples were normalized by sum and each metabolite level was adjusted by autoscaling.

#### Lipidomic profiling

For Lipidomics analysis, 100,000 HK-2 cells were spiked with 2.57 μL of internal standard lipid mixture containing 500 pmol of Chol-d6, 100 pmol of Chol-16:0-d7, 100 pmol of DG 17:0-17:0, 50 pmol of TG 17:0-17:0-17:0, 100 pmol of SM 18:1; 2-12:0, 30 pmol of Cer 18:1; 2-12:0, 30 pmol of GalCer 18:1; 2-12:0, 50 pmol of LacCer 18:1; 2-12:0, 300 pmol of PC 17:0-17:0, 50 pmol of PE 17:0-17:0, 50 pmol of PI 16:0-16:0, 50 pmol of PS 17:0-17:0, 30 pmol of PG 17:0-17:0, 30 pmol of PA 17:0-17:0, 40 pmol of Gb3 18:1; 2-17:0, 25 pmol of GM3 18:1; 2-18:0-d5, 25 pmol of GM2 18:1; 2-18:0-d9, 25 pmol of GM1 18:1; 2-18:0-d5, 30 pmol of LPA 17:0, 30 pmol of LPC 12:0, 30 pmol of LPE 17:1 and 30 pmol of LPS 17:1 and subjected to lipid extraction at 4°C, as described elsewhere.^70^ Briefly, the sample was dissolved in 200 μL of 155 mM ammonium bicarbonate and then extracted with 1 mL of chloroform-methanol (10:1) for 2 h. The lower organic phase was collected, and the aqueous phase was re-extracted with 1 mL of chloroform-methanol (2:1) for 1 h. The lower organic phase was collected and evaporated in a SpeedVac vacuum concentrator. Lipid extracts were dissolved in 100 μL of infusion mixture consisting of 7.5 mM ammonium acetate dissolved in propanol:chloroform:methanol [4:1:2 (vol/vol)]. Samples were analyzed by direct infusion in a QExactive mass spectrometer (Thermo Fisher Scientific) equipped with a TriVersa NanoMate ion source (Advion Biosciences). 5 μL of sample were infused with gas pressure and voltage set to 1.25 psi and 0.95 kV, respectively. DG, TG and CE species were detected in the 10:1 extract, by positive ion mode Fourier transform mass spectrometry (FTMS) as ammonium adducts by scanning m/z = 580–1000 Da, at R_m/z=200_ = 280 000 with lock mass activated at a common background (m/z = 680.48022) for 30 s. Every scan is the average of 2 micro-scans, automatic gain control (AGC) was set to 1E6 and maximum ion injection time was set to 50 ms. For FA profiling of DG and TG, a parallel reaction monitoring was performed with an inclusion list of m/z = 580–1000 Da, at normal collision energy (NCE) of 20 and R_m/z=200_ = 17500 for 90 s. Every scan is the average of 2 micro-scans, automatic gain control was set to 1E5 and maximum ion injection time was set to 64 ms.

PC, PCO, Cer, GlcCer, LPC and LPCO were detected as acetate adducts while PG, PE, PEO, LPE and LPEO were detected as deprotonated adducts in the 10:1 extract, by negative ion mode FTMS, after polarity switch by scanning m/z = 420–1050 Da, at R_m/z=200_ = 280 000 with lock mass activated at a common background (m/z = 529.46262) for 30 s. Every scan is the average of 2 micro-scans, automatic gain control was set to 1E6 and maximum ion injection time was set to 50 ms. For FA profiling of PC, PCO, PE, PEO and PG, a parallel reaction monitoring was performed with an inclusion list of m/z = 590–940 Da, at NCE of 35 and R_m/z=200_ = 17500 for 72 s. Every scan is the average of 2 micro-scans, AGC was set to 1E5 and maximum ion injection time was set to 64 ms.

LacCer and Gb3 were detected as protonated ions and Gb4 was detected as ammoniated adduct in the 2:1 extract in positive ion mode FTMS by scanning m/z = 800-1,600 Da, at R_m/z=200_ = 280,000 with lock mass activated at a common background (m/z = 1,194.8179) for 30 s. GM1, GM2, andGM3 were detected as deprotonated ions in the 2:1 extract in negative ion mode after polarity switch in FTMS by scanning m/z = 1,100–1,650 Da, at R_m/z=200_ = 280,000 with lock mass activated at a common background (m/z = 1,175.7768) for 30 s. Every scan is the average of two micro-scans, AGC was set to 1E6 and IT was set to 50 ms in both polarities.

PA, PI, PS, LPA, and LPS were detected as deprotonated ions in the 2:1 extract in negative ion mode in FTMS by scanning m/z = 400-1,100 Da, at R_m/z=200_ = 280,000 with lock mass activated at a common background (m/z = 529.4626) for 30 s. Every scan is the average of two micro-scans, AGC was set to 1E6 and IT was set to 50 ms. For FA profiling of PA, PI and PS, a parallel reaction monitoring was performed with an inclusion list of m/z = 590–940 Da, at NCE of 35 and R_m/z=200_ = 17500 for 84 s. Every scan is the average of 2 micro-scans, automatic gain control (AGC) was set to 1E5 and maximum ion injection time was set to 64 ms.

All data was acquired in centroid mode. All lipidomics data were analyzed with the lipid identification software, LipidXplorer.^71^ Tolerance for MS and identification was set to 2 ppm. Data post-processing and normalization to internal standards were done manually in Excel. Data analysis was performed in the MetaboAnalyst 5.0 software.^69^ Samples were normalized by sum and each lipid species level was adjusted by autoscaling.

### Quantification and statistical analysis

Graphs and statistical analyses were generated using GraphPad Prism 9 software (GraphPa Software, Inc.). Data are presented as mean ± standard deviation. Unpaired 2-sample t-tests were used to determine a significant difference between two groups and were 2-tailed. Multiple comparisons were performed with one-way ANOVA followed by a Dunnett’s multiple comparison test. Multiple comparisons between groups were performed with two-way ANOVA followed by a multiple comparisons test. The rate of graft failure evaluated using Kaplan-Meier curves, Log-Rank test and a Cox proportional hazards model. A *p*-value < 0.05 was considered a statistically significant difference.

## Supplemental information

Document S1. Figures S1–S4 and Tables S1–S3

Document S2. Raw data for figures
