# Supplementary material for: Interferon gamma induced-ACSL5 shapes the lipidome of kidney tubular cells
Source: iScience. 2025 May 23;28(6):112742. doi: 10.1016/j.isci.2025.112742 (PMC12179626; doi:10.1016/j.isci.2025.112742)
Supplement: Document S1. Figures S1–S4 and Tables S1–S3 [file mmc1.pdf]

iScience, Volume *28*

Supplemental information

Interferon gamma induced-ACSL5 shapes the lipidome of kidney tubular cells

Virginie Poindessous, Julio L. Sampaio, Lydia Bouidghaghen, Ivan Nemazanyy, Alexandre Pallet, Maarten Naesens, Thibaut Vaulet, Dany Anglicheau, and Nicolas Pallet

FIGURE S1

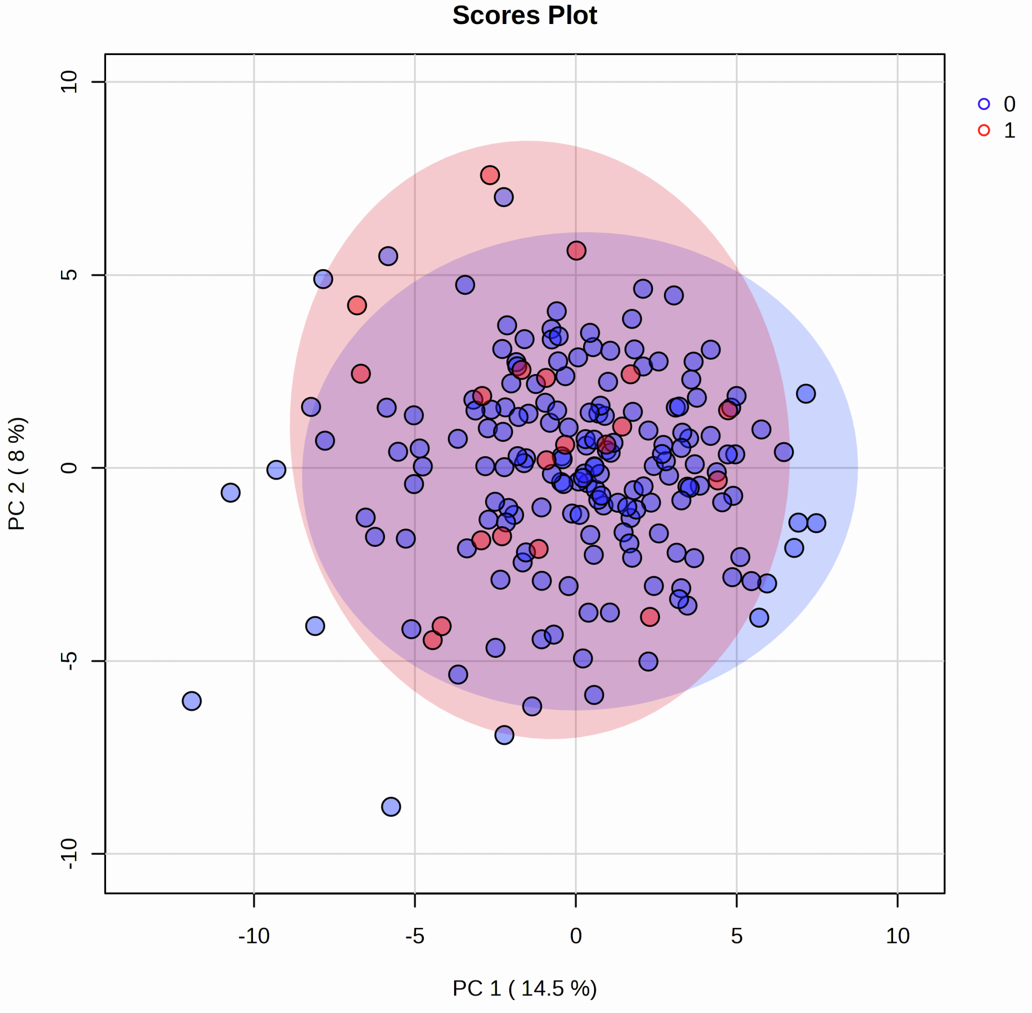

FIGURE S2

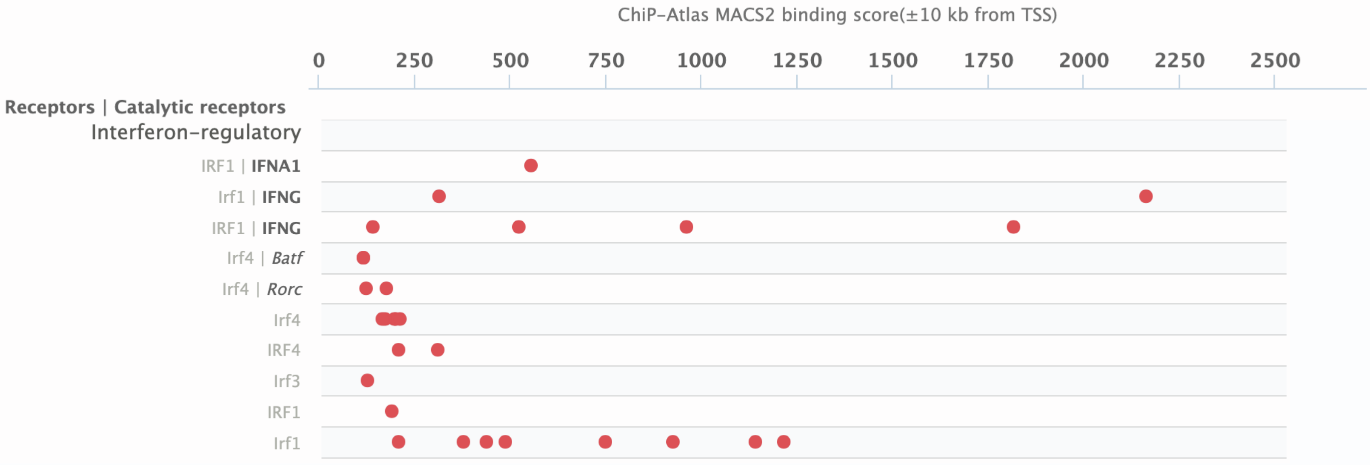
ChiP-Atlas MACS2 binding score(±10 kb from TSS)
0
250
500
750
1000
1250
1500
1750
2000
2250
2500
Receptors | Catalytic receptors
Interferon-regulatory
IRF1 | IFNA1
Irf1 | IFNG
IRF1 | IFNG
Irf4 | Batf
Irf4 | Rorc
Irf4
IRF4
Irf3
IRF1
Irf1

FIGURE S3

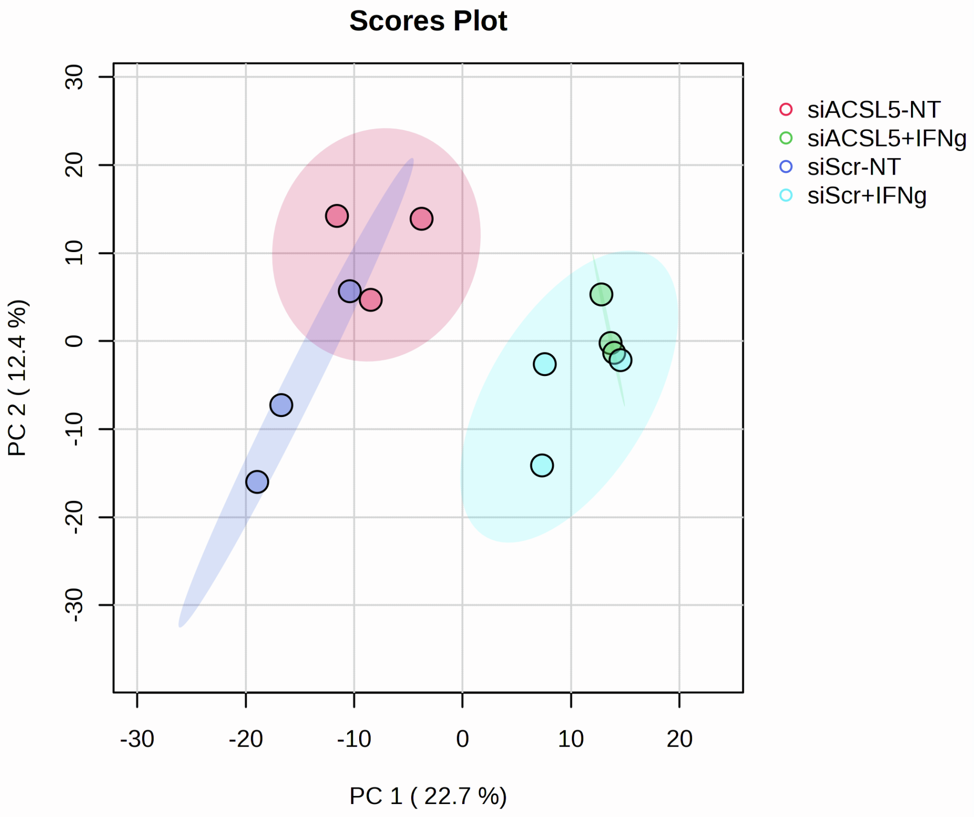
Scores Plot
PC 2 (12.4 %)
PC 1 (22.7 %)
30
20
10
0
-10
-20
-30
-30
-20
-10
0
10
20
siACSL5-NT
siACSL5+IFNg
siScr-NT
siScr+IFNg

FIGURE S4

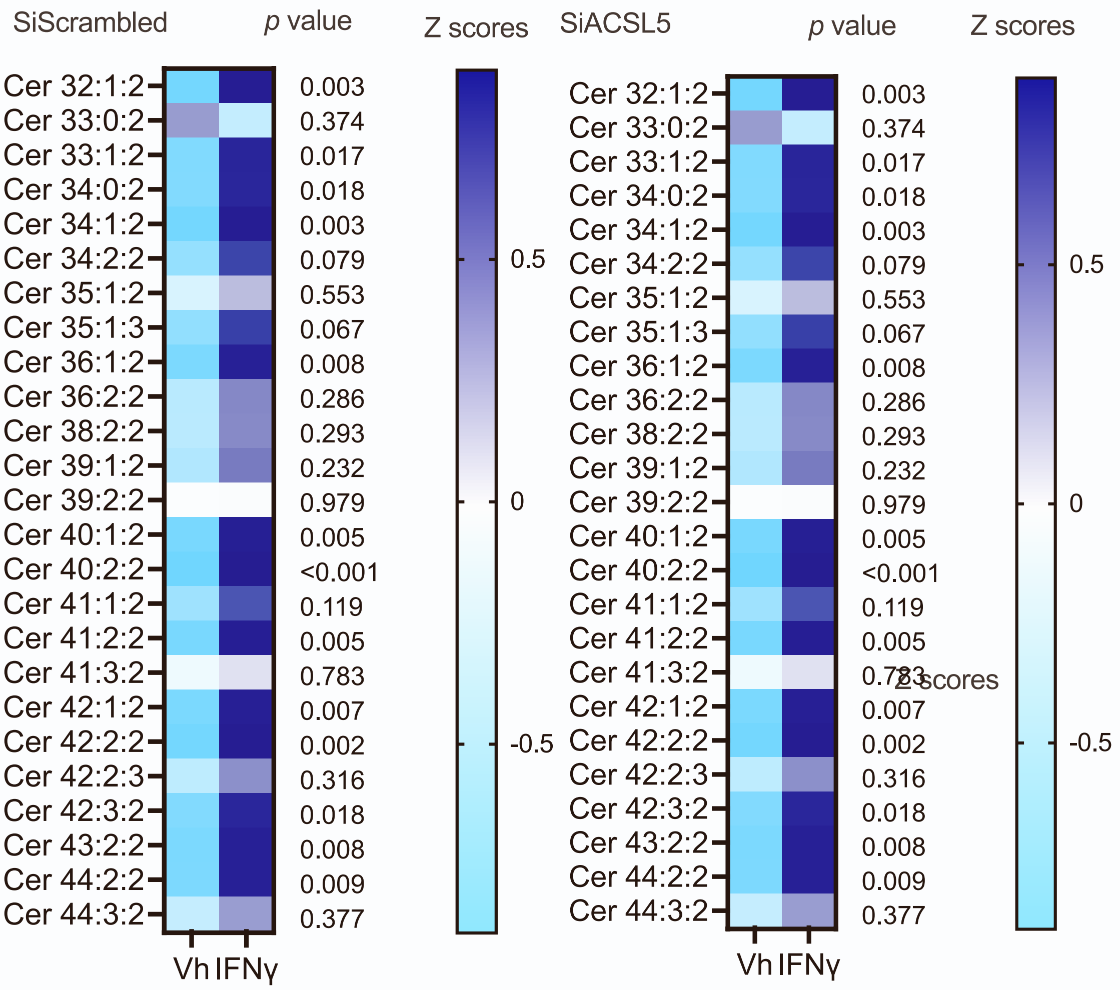
SiScrambled
p value
Z scores
Cer 32:1:2
Cer 33:0:2
Cer 33:1:2
Cer 34:0:2
Cer 34:1:2
Cer 34:2:2
Cer 35:1:2
Cer 35:1:3
Cer 36:1:2
Cer 36:2:2
Cer 38:2:2
Cer 39:1:2
Cer 39:2:2
Cer 40:1:2
Cer 40:2:2
Cer 41:1:2
Cer 41:2:2
Cer 41:3:2
Cer 42:1:2
Cer 42:2:2
Cer 42:2:3
Cer 42:3:2
Cer 43:2:2
Cer 44:2:2
Cer 44:3:2
0.003
0.374
0.017
0.018
0.003
0.079
0.553
0.067
0.008
0.286
0.293
0.232
0.979
0.005
<0.001
0.119
0.005
0.783
0.007
0.002
0.316
0.018
0.008
0.009
0.377
0.5
0
-0.5
Vh
IFNγ
SiACSL5
p value
Z scores
Cer 32:1:2
Cer 33:0:2
Cer 33:1:2
Cer 34:0:2
Cer 34:1:2
Cer 34:2:2
Cer 35:1:2
Cer 35:1:3
Cer 36:1:2
Cer 36:2:2
Cer 38:2:2
Cer 39:1:2
Cer 39:2:2
Cer 40:1:2
Cer 40:2:2
Cer 41:1:2
Cer 41:2:2
Cer 41:3:2
Cer 42:1:2
Cer 42:2:2
Cer 42:2:3
Cer 42:3:2
Cer 43:2:2
Cer 44:2:2
Cer 44:3:2
0.003
0.374
0.017
0.018
0.003
0.079
0.553
0.067
0.008
0.286
0.293
0.232
0.979
0.005
<0.001
0.119
0.005
0.783 Z scores
0.007
0.002
0.316
0.018
0.008
0.009
0.377
0.5
0
-0.5
Vh
IFNγ

SUPPLEMENTARY FIGURES TITLES AND LEGENDS

FIGURE S1. [Principal Component Analysis] related to FIGURE 2

Dimension reduction by Principal Component Analysis (PCA) of all the metabolites the 193 KTR urine samples collected 3 months after kidney transplantation and who underwent of protocol biopsy. The plot shows the 2D scores between the selected PC that best explain the variance of metabolites.

FIGURE S2. [IRF1 binding sites] related to FIGURE 3

Schematic of the IRF1 binding sites in the promoter of human *ACSL5* gene generated using ChiP-Seq datasets integrated and analysed by the Signalling Pathways Project, a multi-omics knowledge mine based upon public, manually curated transcriptomic and cistromic (ChIP-Seq) datasets. http://www.signalingpathways.org/.

FIGURE S3. [Principal Component Analysis] related to FIGURE 5

Dimension reduction by Principal Component Analysis (PCA) of all the lipid species identified in HK2 cells after transfection with control siRNA (siScr) or with siRNA directed against *ACLS5* RNA (siACSL5) and incubated with 100 mg/ml IFNγ or vehicle (Vh, water) for 48 hours (n=3). The plot shows the 2D scores between the selected PC that best explain the variance of lipids

FIGURE S4. [Relative composition in ceramides] related to FIGURE 5

Heat map showing the relative composition (mean z-score) in ceramide molecular species identified in HK2 cells after transfection with control siRNA (**A**) (siScr) or with siRNA directed against *ACLS5* RNA (siACSL5) (**B**) and incubated with 100 mg/ml IFNg or Vehicle (water) for 48 hours (n=3). Each line corresponds to the proportional composition in lipid class after autoscaling (i.e., normalizing the data by centering the mean and dividing by the standard deviation of each variable).

SUPPLEMENTARY TABLES

Supplementary Table S1. Demographic and clinical characteristics of the cohort of 193 kidney transplant recipients who had an urinary metabolome profiling 3 months after transplantation.

**Characteristics**	**Entire cohort** (n=193)
Age (years)	53±20
Male sex-n (%)	115 (59)
Cause of ESRD-n (%)	
• GN	55 (20)
• Diabetes	38 (14)
• Cystic/hereditary	54 (20)
• Secondary GN	23 (8.5)
• Hypertension	18 (6)
• Interstitial nephritis	18 (6)
• Miscellaneous	10 (3)
• Uncertain	56 (20)
Donor age (years)	50 (15)
Living donor-n (%)	51 (26)
Expanded criteria donor-n (%)	119 (35)
Retransplantation-n (%)	27 (14)
Preformed DSA-n (%)	88 (45)
Cold ischemia time (hours)	14.8±11
Delayed graft function-n (%)	44 (22)
Plasma creatinine at month 3 (μmol/L)	140±51

ESRD: End Stage Renal Disease

GN: glomerulonephritis

DSA: donor specific antibodies

Supplementary Table S2. Differentially Eexpressed Genes in non-injured versus injured proximal tubules; No rejection group.

	p_val	avg_log2FC	p_val_adj
**ACSL3**	1.78E-50	-1.9478756	7.16E-46
**ACSL4**	4.69E-48	-1.1810961	1.89E-43
**ACSL1**	1.66E-41	0.54915088	6.69E-37
**SLC27A2**	3.17E-38	0.90696108	1.28E-33
**ACSF2**	3.59E-37	1.55360892	1.45E-32
**ACSM3**	5.33E-33	1.20960284	2.15E-28
**ACSS1**	1.34E-31	-1.1710717	5.41E-27
**ACSS2**	8.30E-31	0.56153353	3.35E-26
**ACSM2A**	1.38E-30	-0.3975435	5.56E-26
**ACSM2B**	3.03E-27	0.29811857	1.22E-22
**AACS**	6.09E-24	-0.7233427	2.46E-19
**ACSF3**	3.13E-23	-0.2090623	1.26E-18
**ACSM5**	2.80E-21	1.10284615	1.13E-16
**ACSL5**	1.86E-20	-3.4606924	7.49E-16
**ACSS3**	2.23E-18	0.16968599	8.98E-14
**ACSM1**	3.41E-06	-1.4358882	0.137574927

Supplementary Table S3. Differentially Eexpressed Genes in non-injured versus injured proximal tubules; Rejection group.

	p_val	avg_log2FC	p_val_adj
**ACSF2**	1.56E-77	1.397891	6.30E-73
**ACSL4**	9.76E-74	-1.08532	3.94E-69
**ACSM2B**	1.03E-65	0.056089	4.17E-61
**ACSL3**	1.81E-56	-1.17588	7.29E-52
**ACSL1**	2.13E-52	0.937985	8.60E-48
**ACSM2A**	7.29E-47	-0.57871	2.94E-42
**ACSS2**	5.20E-46	0.506655	2.10E-41
**ACSS1**	4.25E-38	-0.85812	1.72E-33
**SLC27A2**	1.69E-36	1.125696	6.83E-32
**ACSL5**	8.07E-32	-2.80794	3.26E-27
**AACS**	3.73E-31	-0.21401	1.51E-26
**ACSS3**	1.74E-30	0.528177	7.04E-26
**ACSM3**	8.43E-28	1.172081	3.40E-23
**ACSF3**	3.90E-27	-0.21334	1.58E-22
**ACSM5**	4.05E-21	1.189556	1.63E-16
**ACSM1**	5.76E-17	-0.68795	2.32E-12
